# Plug-flow hydrolysis with lignocellulosic residues: effect of hydraulic retention time and thin-sludge recirculation

**DOI:** 10.1186/s13068-023-02363-7

**Published:** 2023-07-06

**Authors:** Theresa Menzel, Peter Neubauer, Stefan Junne

**Affiliations:** 1 Chair of Bioprocess Engineering, Institute of Biotechnology, Technische Universität Berlin, Ackerstraße 76, ACK 24, 13355 Berlin, Germany; 2 Department of Chemistry and Bioscience, Aalborg University Esbjerg, Niels Bohrs Vej 8, 6700 Esbjerg, Denmark

**Keywords:** Microbial hydrolysis, Plug-flow reactor, Anaerobic digestion, Recirculation, Hydraulic retention time

## Abstract

**Background:**

Two parallel plug-flow reactors were successfully applied as a hydrolysis stage for the anaerobic pre-digestion of maize silage and recalcitrant bedding straw (30% and 66% w/w) under variations of the hydraulic retention time (HRT) and thin-sludge recirculation.

**Results:**

The study proved that the hydrolysis rate profits from shorter HRTs while the hydrolysis yield remained similar and was limited by a low pH-value with values of 264–310 and 180–200 g_O2_ kg_VS_^−1^ for 30% and 66% of bedding straw correspondingly. Longer HRT led to metabolite accumulation, significantly increased gas production, a higher acid production rate and a 10–18% higher acid yield of 78 g_SCCA_ kg_VS_^−1^ for 66% of straw. Thin-sludge recirculation increased the acid yield and stabilized the process, especially at a short HRT. Hydrolysis efficiency can thus be improved by shorter HRT, whereas the acidogenic process performance is increased by longer HRT and thin-sludge recirculation. Two main fermentation patterns of the acidogenic community were found: above a pH-value of 3.8, butyric and acetic acid were the main products, while below a pH-value of 3.5, lactic, acetic and succinic acid were mainly accumulating. During plug-flow digestion with recirculation, at low pH-values, butyric acid remained high compared to all other acids. Both fermentation patterns had virtually equal yields of hydrolysis and acidogenesis and showed good reproducibility among the parallel reactor operation.

**Conclusions:**

The suitable combination of HRT and thin-sludge recirculation proved to be useful in a plug-flow hydrolysis as primary stage in biorefinery systems with the benefits of a wider feedstock spectrum including feedstock with cellulolytic components at an increased process robustness against changes in the feedstock composition.

**Graphical Abstract:**

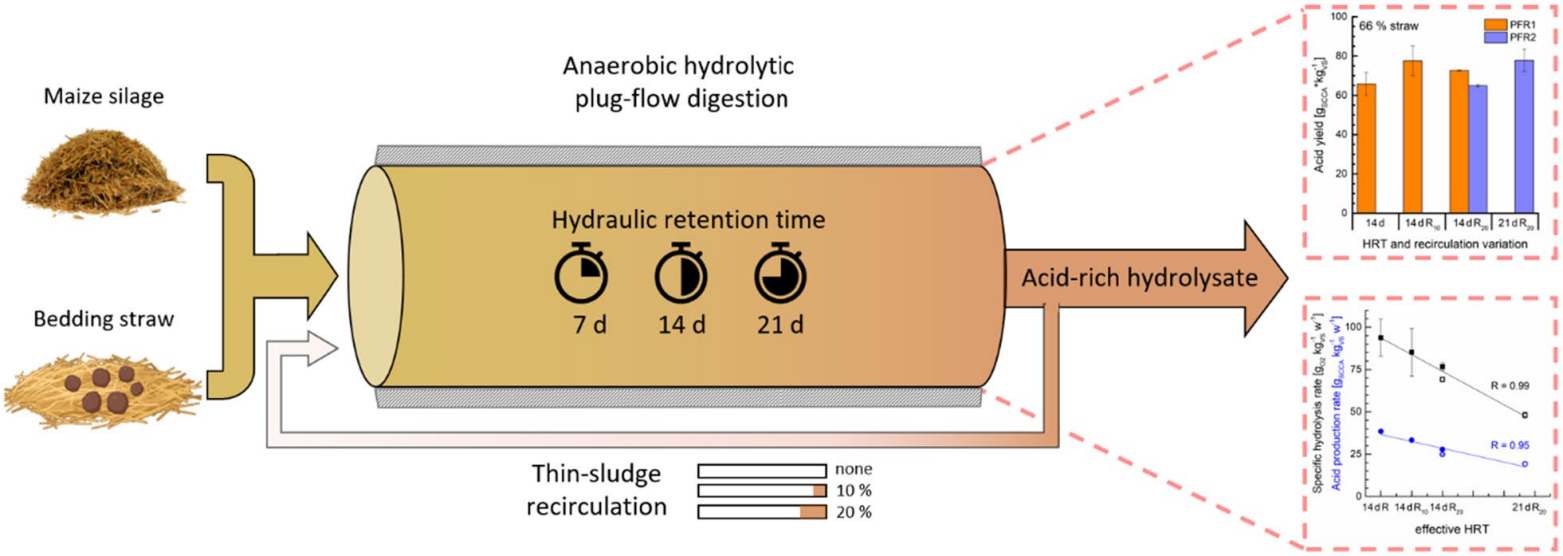

**Supplementary Information:**

The online version contains supplementary material available at 10.1186/s13068-023-02363-7.

## Highlights

- Continuous hydrolysis of lignocellulosic residues was performed in plug-flow reactors without pretreatment or pH-control
- The impact of HRT and recirculation on hydrolytic and acidogenic conversion efficiency is discussed
- Longer HRT and thin-sludge recirculation led to higher acid yields from recalcitrant feedstock
- Different valuable acids (butyric/acetic/lactic acid) can be produced with virtually equal yields in this hydrolysis stage

**Supplementary Information:**

The online version contains supplementary material available at 10.1186/s13068-023-02363-7.

## Background

Biogenic residues represent an enormous potential for the production of energy and valuable products in a circular bioeconomy. Residues like the organic fraction of municipal waste, food waste, agricultural residues and wastewater, among others, are already used for the production of biogas via anaerobic digestion (AD). Most often, however, hydrolysis of the feedstock is not complete or requires a long time. In order to increase the flexibilization of biomass use and direct the industry towards a more decentralized and dynamic application of feedstock, the achievement of a versatile hydrolysis is of high importance. In particular, with lignocellulosic residues, often extensive and tailor-made pretreatments are necessary to gain a sufficient hydrolysis efficiency. Two-stage AD, in which the hydrolysis/acidogenesis process is separated from the acetogenesis/methanogenesis step has already been shown to increase process stability: the first hydrolytic stage is usually robust against a change of the feedstock mixture and quality, which facilitates the practical operation and control of large-scale AD. This setup is often combined with thin-sludge recirculation from the second to the first stage, which has been shown to significantly increase process efficiency and stability [1]. Recirculation improves nutrient availability in the first reactor [2–4], stabilizes the pH-value due to the higher buffer capacity of the methanogenic phase [1, 4–7], enhances mass transfer especially in dry AD systems like leach bed reactors [2, 8] and can enrich key microorganisms involved in hydrolysis and acidogenesis [8, 9]. However, there is a lack of research on the effect of recirculation within the hydrolysis/acidogenesis phase itself. Dong et al*.* [9] examined the digestion of cattle manure in a plug-flow reactor (PFR) with the application of 50% recirculation within the acidic phase. They found higher biogas production, digestion efficiency and, most importantly, an enrichment of hydrolytic bacteria in the front part of the reactor, while microorganism involved in the recalcitrant cellulose-digestion increased in the back part of the reactor.

The operation in a PFR, instead of the frequently applied stirred tank reactors, has several characteristic features: (1) gradient formation in the reactor allows the establishment of microenvironments for specialized microbes [10, 11]; (2) operation at a high total solids content [12]; (3) low energy input for stirring [13, 14]. Research on the acidic digestion of lignocellulosic residues in PFRs is rare, although it holds big potential for industrial applications. By the knowledge of the authors, large-scale PFRs with recirculation are already applied; however, the impact of feedstock variation or process conditions on the hydrolysis efficiency and the microbial community are not well understood in PFR systems.

Anaerobic microbial hydrolysis is facilitated through the production of exoenzymes and membrane-bound enzymes. These enzymes penetrate and degrade the polymeric molecules to oligo- and monomeric substances like sugars, amino acids or fatty acids. These molecules can then be converted to various short-chain carboxylic acids (SCCAs) through acidogenic bacteria. The conversion naturally depends on substrate availability, pH-value, oxidation–reduction potential (ORP), microbial community structure and hydrogen partial pressure, among others [15–18]. A pH-value between 5.0 and 6.3 and an ORP value of about – 300 mV have been associated with a major production of acetic and butyric acid [13, 16, 17]. A pH-value below 4.0 usually leads to the dominance of lactic acid bacteria, which produce mainly lactic acid and ethanol [17, 19]. A high partial pressure of hydrogen can favor lactic and propionic acid production [16]. High recirculation of 50–75% [2] and a higher pH-value between 7.7 and 8.3 [20] were found to increase the production of *iso*-/valeric acid. Generally, longer HRT have been found to improve hydrolysis [21] and acidogenesis yield and shift the SCCA spectrum towards longer and higher value acids as butyric or caproic acid [22–24]. Nonetheless, the HRT has to be optimized to achieve the highest possible throughput of feedstock combined with a good digestion efficiency [25].

We hypothesize that by using a PFR with thin-sludge recirculation, efficient hydrolysis of recalcitrant feedstock is feasible without further pretreatment. To examine the impact of process conditions and feedstock variation on the hydrolysis efficiency, the hydrolytic digestion of maize silage as reference was mixed with an increasing content of bedding straw at various HRTs and with changing recirculation patterns. Usually, in continuous stirred tank reactors, the evaluation of new steady-state conditions is only applicable after at least 3 HRTs have passed [26]. However, the plug-flow regime should ideally lead to an exchange of the whole reactor material after 1 HRT, making it possible to evaluate a process state (most often in a quasi-steady-state mode close to a true steady state) after 2–3 HRTs already. This was applied in our study in order to achieve several conditions in the observation period.

Straw is an abundant lignocellulosic feedstock with high carbon, but low nitrogen content. Wheat straw has been estimated to have a yearly unused energy potential of 373 PJ for biohydrogen production worldwide [27], so exploitation of this biogenic resource, including the application in acidogenic fermentation, is of high interest. To overcome its bad digestibility due to the lignocellulosic structure, it is common to apply various pretreatment methods like acidic or alkali pretreatment [28–30], enzymatic treatments [31], milling [32, 33] or thermo-oxidation with H_2_O_2_ [34], among others. Another method is the fermentation of straw in co-digestion with manure, which offers many advantages as it provides an improved C/N ratio, better nutrient balance, dilution of toxic substances, and thus a higher biogas yield [28, 35–37]. However, hydrolysis or acid yields from the anaerobic microbial hydrolysis of straw are rarely described in literature. In contrast, MS is a common substrate for AD. Biogas, hydrogen or acid yields have been described for numerous process conditions and reactor types, making the substrate quite suitable for a comparison of efficiencies.

The aim of this research was then to (1) investigate the effect of HRT and thin-sludge recirculation on hydrolysis and acidogenesis efficiency; (2) determine hydrolysis efficiency in the single-stage PFR at different feedstock composition with an increasing content of bedding straw; (3) compare all data for the identification of most suitable operation conditions at the individual feedstock compositions for the application as hydrolysis stage.

## Material and methods

### Feedstock characteristics

The feedstock used in this study was whole plant maize silage (MS) from a farm in Bavaria, Germany, and bedding straw from a horse stable from uFA Fabrik Berlin, Germany. Upon arrival at our lab, both feedstocks were dried and sieved with a particle size of ≤ 0.5 cm. Larger particles were crushed with a blender, until the desired size was achieved. The bedding straw contained, among different straw varieties (wheat and oat, among others) also horse manure, residues of feed (grains, apples), and sand in small amounts. Inert materials like stones, plastics and the majority of sand were taken out before digestion. Samples of the feedstock were milled and characterized. As the operation of the PFRs lasted over 1.5 years, different feedstock batches were applied. Each feedstock batch was analyzed and correspondingly used for calculations of its load. In Table 1, the average values of the features of the feedstock are depicted. The methods used for characterization of the feedstock have been described elsewhere [38].

**Table 1:** Average substrate characteristics of maize silage and bedding straw

Parameter	Unit	Maize silage		Bedding straw	
		Ave	SD	Ave	SD
pH-value		4.64	1.07	7.63	0.72
TS	%	29.12	3.41	/	/
Moisture	%	70.88	3.41	/	/
VS	% of TS	96.85	0.70	83.54	8.38
Ashes	% of TS	3.15	0.70	20.36	13.06
tCOD	gO_2_ g_TS_^−1^	0.85	0.03	0.50	0.27
sCOD	gO_2_ g_TS_^−1^	0.13	0.03	0.06	0.02
Total nitrogen	% of TS	0.95	0.08	0.62	0.17
Non-structural carb. content	% of TS	31.60	8.68	12.77	4.72
Acid-insoluble lignin	% of TS	15.07	1.70	29.19	2.34
Acid-soluble lignin	% of TS	1.48	0.43	1.35	0.40

Characterization has been done for different substrate batches over time (see Additional file 1: Table S3)

### Operation of parallel reactor experiments

Two identically designed PFRs (FWE GmbH, Germany) were operated continuously over 1.5 years in parallel with varying conditions of the HRT and thin-sludge recirculation to examine the effects of operation on the acidic AD of MS mixed with bedding straw. These two reactors are referred to as PFR1 and PFR2 throughout this manuscript. The operation of PFR1 concerning a bioaugmentation with *Paenibacillus* spp. has been described in detail previously [38]. Briefly described, the start of both processes was performed by inoculation from the effluent from a dark fermentation in a similar PFR operated with MS as feedstock at pH 4.0–4.2. MS, the inoculum (up to 3 L) and water were added to achieve a TS content of approx. 4–6% in week 1. In between weeks 2 and 4, the organic loading rate was kept between 3.2 and 4.5 kg_VS_ m^−3^ d^−1^ in order to achieve a working volume of between 12 and 13 L with about 10% TS content by week 4 in both reactors. The HRT was gradually reduced in between weeks 2 and 4, so that an HRT of 14 d was achieved by week 4 (respectively 5 in PFR2). The pH-value was adjusted with 30% NaOH once in week 4 (5 in PFR2) to prevent acidification below a pH-value of 4.0. No further control of the pH-value was applied as a stable microbial community, characterized by a steady acid and gas profile, developed over time in both reactors. The OLR was held at 4 kg_VS_ m^−3^ d^−1^ throughout the cultivation, whereas the HRT and recirculation of thin-sludge from the end to the inlet tube of the reactor were changed dynamically as shown in Table 2. Due to a technical failure of the hardware, the process in PFR2 had to be stopped after 16 weeks of operation. The reactor liquid and digestate were frozen and used as inoculum for the restart of the reactor. PFR2 was restarted using 4.21 L inoculum—reactivated at 36 °C for 24 h, 2.3 kg MS digestate (12.2% TS), 6.2 L H_2_O, 125 g dry MS and 110 g dry straw in week 20, so that a working volume of 12.5 L was achieved. In week 21, continuous operation was started with 14 d of HRT and 4 kg_VS_ m^−3^ d^−1^ OLR, with 10% TS reached by week 23. No further pH adjustment was conducted. Stirring in PFR2 was stopped for 4 days in week 41 due to technical issues, without any lasting effects on the cultivation.

**Table 2:** Dynamic operation conditions of the PFRs with 3 types of feedstock composition, variations of the HRT and thin-sludge recirculation

Substrate	HRT [d]	Recirculation	Total solids [%]	Period of operation^a^ [w]	Reference period [w]	Loss factor A
**PFR1**						
Maize silage	Start-up	/	n.d	1–3	/	0.85
	7	/ 20%	8.2 (0.4)	9–11 12–14	11 14	
	14	/ / 20%	10.6 (0.8)	4–8 15–16 27–30 16–26^b^	6–8 16 28–30 /	
			12.5 (0.3) /			
30% straw, MS	14	20%	12.3 (0.2) /	31–35.5 36–46^b^	33–35 /	0.75
66% straw, MS	14	20%	14.1 (0.2) /	47–54 55–69^b^	53/54 /	
		10% 0%	13.5 (0.4)	70–74 75–78	73/74 77/78	
**PFR2**						
Maize silage	Start-up	/	n.d	1–4	/	0.85
	14	/ 20%	12.7 (1.0) 14.4 (0.2)	5–8 9–13	7/8 12/13	
*Process in PFR2 stopped. Restart after repair*						
30% straw, MS	Fed-Batch	/	n.d	20	/	0.82
	14 21	20%	10.7 (0.3) 11.1 (0.3)	21–26 27–32	25/26 31/32	
66% straw, MS	21 14	20%	12.0 (0.8) 13.6 (0.5)	34–45 46–51	41–45 50/51	0.96

The data of the reference period are used to compare process efficiency between conditions

^a^Not in chronological order, ^b^Bioaugmentation period, described in [38]

Both reactors were operated in a continuous mode with feeding/harvesting 4 times per week. Three different feedstock mixtures were applied: (1) MS, (2) 30% of bedding straw (w/w) mixed with MS, and (3) 66% of bedding straw (w/w) mixed with MS, respectively. Different operation conditions were held for between 2 and 3 HRTs for the evaluation of their effects on the efficiency of hydrolytic digestion in the PFR. Both reactors were temperature-controlled at mesophilic conditions by external hose-heating and insulation. The reactors were equipped with sensors for the *on-line* measurement of the pH-value, conductivity, ORP and temperature at three locations along the reactor, namely at the inlet, center and outlet part (see [38]). In the present study, the focus was put on the examination of process efficiency under the different operation conditions. The advantages of *on-line* gradient monitoring in between the three locations along the PFR will be presented elsewhere. Samples were taken twice per week at each port. PFR2 was further equipped with an *on-line* gas measurement system for CO_2_, H_2_ and CH_4_ (BCP-BlueSens GmbH, Germany) and a mass flow meter (Vcount, BlueSens) at a tube from the headspace. All *on-line* measurements were set to record every 10 min. Stirring of 5 rpm was applied for laminar mixing. The TS content of both reactors was held between 10 and 13% and determined by weekly mass flow calculations as depicted in Eqs. 1 and 2:

1 $${\text{TS}}_{{\text{PFR}}} \left[ {{\% }} \right] = \frac{{m_{{\text{solids }}\left( {{\text{PFR}}} \right)} }}{{m_{{\text{total}}\left( {{\text{PFR}}} \right)} }}{\cdot}100,$$

2 $$\frac{{A{\cdot}m_{{\text{Feed}}} \left[ {{\text{kg}}} \right] - m_{{\text{Harvest}}} \left[ {{\text{kg}}} \right] - c_{{\text{Harvest}}} \left[ {\frac{{{\text{kg}}}}{{\text{L}}}} \right]{\cdot}V_{{\text{Harvest}}} \left[ {\text{L}} \right]}}{{m_{{\text{solids}}} \left[ {{\text{kg}}} \right] + m_{{\text{H}}_2 {\text{O}}} [{\text{kg}}]}}{\cdot}100.$$

The harvest of the PFR was sieved and its total dry mass (*m*_Harvest_) determined by TS analysis of samples. The solid content of the liquid harvest (*c*_Harvest_) was measured regularly (twice per month) to determine the total mass outflow. The average mass in- and out-flows were determined for each substrate and under different conditions. Their difference was used to calculate the ‘mass loss factor’ *A*. Mass loss in the outflow is due to solubilization and gasification of the feedstock and needs to be accounted for to determine the solids concentration in the reactor.

### Analytical methods

*Off-line* analytics were conducted for the determination of soluble COD (sCOD) and SCCA [38]. The frequency-dispersed anisotropy polarizability (FDAP) was measured *at-line* with Elotrace (EloSystems GbR, Germany) separately for each port as described previously [38]. The scale coefficient of 5 × 10^−31^ F m^2^ was not included in the presentation of the FDAP measurements. The concentration of total reducing sugars was measured with the Nelson–Somogyi method in sterile-filtered samples in duplicates, with glucose used for calibration [39]. The measurement of reducing sugars in both reactors showed only minor differences between samples from the different ports during week 1–16. Accordingly, reducing sugars were only determined at the center port thereafter.

### Metabolic conversion rates

Metabolic conversion of the hydrolytic and acidogenic communities was calculated using the measurements of soluble and total COD and the theoretical COD of the produced SCCA with Eqs. 3–12 [38]. The weekly sCOD and SCCA release were calculated by mass balance including the recirculation (Eqs. 4, 7). The specific rates of hydrolysis and acidogenesis (Eqs. 5, 9) were calculated using the dilution rate (*D*). *D* was calculated by the total inflow (*F*) subtracted by the recirculation flow (*R*), divided by the reaction volume (*V*, Eq. 10). To determine the acidification (Eq. 12), the theoretical COD of the SCCA was calculated with Eq. 11 with the COD conversion coefficients (g_COD_ g_SCCA_^−1^) of 1.07 (acetic/lactic acid), 1.82 (butyric acid), 1.51 (propionic acid), 0.95 (succinic acid), 0.91 (pyruvic acid), 0.75 (citric acid), 2.04 (valeric acid) and 2.20 (caproic acid) [2]:

3 $${\text{Hydrolysis }}\left[ {{\% }} \right] = \frac{{s{\text{COD }}\left( t \right)}}{{t{\text{COD }}\left( t \right)}},$$

4 $${\mathop {s{\text{COD}}}\limits^{\cdot}}_{{\text{released}}} { }\left[ {\frac{{{\text{gO}}_2 }}{w}} \right] = \dot{s\text{COD}}_{out}-\dot{\text{COD}}_{in}-\dot{\text{COD}}_{Recirc},$$

5 $${\text{Specific}}\,{\text{hydrolysis}}\,{\text{yield}}\,\left( {{\text{SHY}}} \right){ }\left[ {\frac{{{\text{gO}}_{2{\text{released}}} }}{{{\text{k}}g_{{\text{VS}}\,{\text{fed}}} }}} \right] = \frac{{{\mathop {s{\text{COD}}}\limits^{\cdot}}_{{\text{released}}} }}{{{\text{VS}}_{{\text{in}}} }},{ }$$

6 $${\text{Specific}}\,{\text{hydrolysis}}\,{\text{rate}}\,\left( {{\text{SHR}}} \right){ }\left[ {\frac{{{\text{gO}}_{2{\text{released}}} }}{{{\text{kg}}_{{\text{VS}}\,{\text{fed}}} \cdot w}}} \right] = \frac{{{\mathop {s{\text{COD}}}\limits^{\cdot}}_{{\text{released}}} }}{{{\text{VS}}_{{\text{in}}} }}{*}D,{ }$$

7 $${\mathop {{\text{SCCA}}}\limits^{\cdot}}_{{\text{released}}} { }\left[ {\frac{{g_{{\text{SCCA}}} }}{w}} \right] = {\dot{\text{SCCA}}}_{{\text{out}}} - {\dot{\text{SCCA}}}_{{\text{Recirc}}} ,$$

8 $${\text{Acid}}\,{\text{yield}}\, \left( {{\text{AY}}} \right){ }\left[ {\frac{{{\text{g}}_{{\text{SCCA}}} }}{{{\text{kg}}_{{\text{VS}}\,{\text{fed}}} }}} \right] = \frac{{{\mathop {{\text{SCCA}}}\limits^{\cdot}}_{{\text{released}}} }}{{{\text{VS}}_{{\text{in}}} }},$$

9 $${\text{Acid}}\,{\text{production}}\,{\text{rate}}\, \left( {{\text{APR}}} \right){ }\left[ {\frac{{{\text{g}}_{{\text{SCCA}}} }}{{{\text{kg}}_{{\text{VS}}\,{\text{fed}}} \cdot w}}} \right] = \frac{{{\mathop {{\text{SCCA}}}\limits^{\cdot}}_{{\text{released}}} }}{{{\text{VS}}_{{\text{in}}} }}*D,$$

10 $$D = \frac{F - R}{{V_{{\text{PFR}}} }},$$

11 $${\text{COD}}_{{\text{SCCA}}} = c_{{\text{Acid}}} \left[ {\frac{{{\text{g}}_{{\text{SCCA}}} }}{L}} \right]\cdot{\text{conversion}}\,{\text{coefficient}} \left[ {\frac{{{\text{g}}_{{\text{COD}}} }}{{{\text{g}}_{{\text{SCCA}}} }}} \right],$$

12 $${\text{Acidification }}\left[ {{\% }} \right] = \frac{{{\text{COD}}_{{\text{SCCA}}} (t)}}{{s{\text{COD }}(t)}} _.$$

## Results

In order to study the effects of various HRTs and thin-sludge recirculation conditions and benefits for the microbial hydrolysis of residual lignocellulosic feedstock, in this case bedding straw, two identical PFR have been operated for approx. 1.5 years under dynamic conditions with varying feedstock mixtures. Different operation conditions were maintained for between 2 and 3 HRTs to achieve a quasi-steady state that allows the evaluation of hydrolysis and acidogenesis efficiency depending on the process operation. *Off-line* measurements as obtained after 2–3 HRTs are shown in Table 3 for all conditions.

**Table 3:** Average quasi-steady-state conditions in both PFRs depending on feedstock and process conditions

Feedstock	Condition	Reactor	sCOD	SCCA	Acetic acid	Butyric acid	Lactic acid	Hydrolysis	Acidification	SHY	Acid yield
			g_O2_ L^−1^	g_SCCA_ L^−1^	%	%	%	%	%	g_O2_ kg_VS_^−1^	g_SCCA_ kg_VS_^−1^
MS	7 d	PFR1	13.5	5.42	21.0 ± 0.3	71.1 ± 1.0	1.56 ± 0.5	17.2	65.4	431.7^a^	173.9
	7 d R_20_		15.1	7.2	22.1 ± 1.8	62.2 ± 3.3	2.22 ± 0.9	19.0	73.8	395.1	185.0
	14 d	PFR1^a^	25.8 ± 3.1	13.0 ± 1.8	17.0 ± 1.3	72.5 ± 1.4	1.91 ± 1.0	23.5 ± 1.9	81.0 ± 3.7	387.5 ± 67.0	203.1 ± 26.6
	14 d R_20_	PFR1	22.1 ± 2.0	8.42 ± 1.4	26.0 ± 0.7	68.0 ± 3.7	0.60 ± 0.15	19.0 ± 1.5	60.0 ± 5.9	218.5 ± 33.2	112.1 ± 22.9
		PFR2	30.1 ± 1.0	13.7 ± 0.1	18.4 ± 0.2	70.4 ± 0.2	1.68 ± 0.0	22.2 ± 1.1	73.3 ± 2.7	377.2 ± 14.4	148.0 ± 2.0
30% straw		PFR1	20.2 ± 2.2 24.5 ± 1.2	7.36 ± 0.4 7.90 ± 0.6	39.6 ± 0.2 25.9 ± 2.7	57.2 ± 0.3 8.1 ± 2.0	0.70 ± 0.3 42.1 ± 8.2	20.2 ± 1.8 31.6 ± 0.4	55.3 ± 3.5 36.0 ± 0.7	264.2 ± 26.6 310.0 ± 6.4^b^	97.2 ± 4.9 101.3 ± 4.2^b^
		PFR2									
66% straw		PFR1	13.8 ± 0.4 14.6 ± 0.2	5.03 ± 0.0 5.22 ± 0.0	37.3 ± 0.2 48.2 ± 0.9	51.4 ± 0.8 37.5 ± 0.3	0.42 ± 0.0 2.2 ± 0.7	16.0 ± 0.4 18.6 ± 0.4	56.0 ± 1.6 50.3 ± 0.8	200.8 ± 5.5 180.9 ± 3.0	72.6 ± 0.4 64.9 ± 0.6
		PFR2									
	14 d R_10_	PFR1	11.5 ± 1.5	4.6 ± 0.4	53.5 ± 0.3	33.1 ± 0.4	2.3 ± 1.0	11.4 ± 1.5	55.4 ± 1.9	195.1 ± 23.0	77.6 ± 7.6
	14 d		9.5 ± 0.3	3.6 ± 0.3	57.5 ± 0.3	29.8 ± 0.4	1.4 ± 0.1	9.4 ± 0.2	51.5 ± 5.9	192.9 ± 20.8	65.9 ± 5.8
30% straw	21 d R_20_	PFR2	35.1 ± 3.4	10.8 ± 0.8	45.6 ± 3.0	2.9 ± 0.1	38.5 ± 1.2	40.2 ± 2.6	33.7 ± 0.9	289.6 ± 21.7^b^	89.1 ± 3.6^b^
66% straw			21.4 ± 1.0	8.52 ± 0.4	35.5 ± 4.2	48.2 ± 7.6	2.1 ± 1.4	28.9 ± 2.5	58.8 ± 2.9	194.5 ± 11.1	77.8 ± 5.7

*R*_20_—20% thin-sludge recirculation, *R*_10_—10% thin-sludge recirculation

^a^No quasi-steady-state reached; ^b^lactic acid fermentation

### Maize silage digestion

MS was digested in the PFR system as a reference feedstock. In order to determine the effect of HRT and recirculation on the hydrolytic digestion of MS, MS was digested with two different HRTs, namely 7 d (with and w/o 20% recirculation) and 14 d in PFR1, while the effect of 20% thin-sludge recirculation at 14 d HRT was examined in PFR2.

**Fig. 1:**
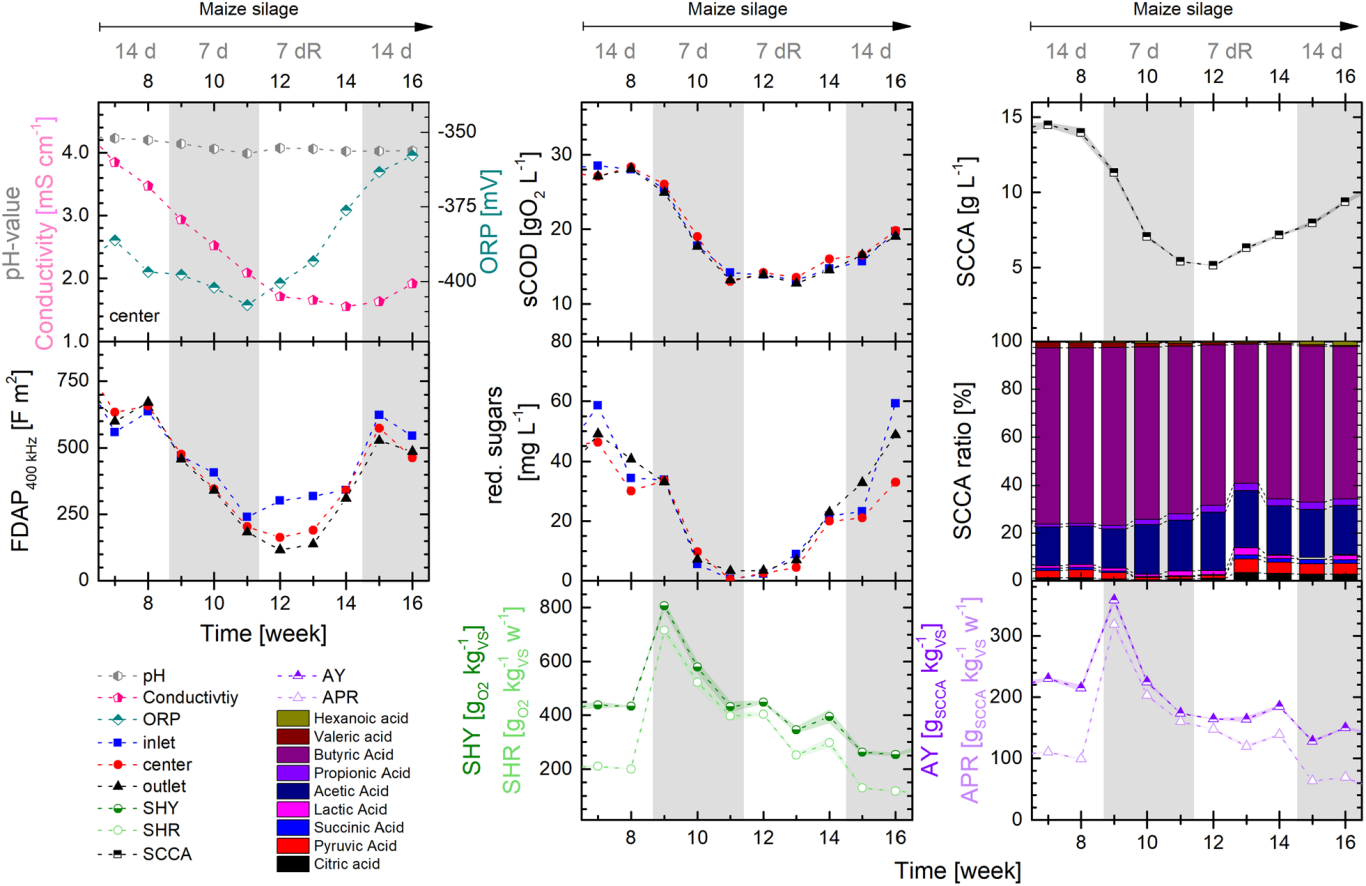
Process conditions in PFR1 operated with MS at different HRTs (14 d / 7 d) and recirculation (*R* = 20% recirculation). Left: *on-line* monitoring of the pH-value, conductivity and ORP measured at the center of the PFR, FDAP measured at 400 kHz. Center: measurements of sCOD, total reducing sugars, SHY and SHR. Right: total SCCA concentration, composition of the SCCA fraction, AY and APR. Depicted are the average values of a week from *on-line/off-line* measurements for each sample and measurement port of the PFR (inlet, center, outlet) or the average values over all ports with shaded deviations in-between ports (SHR, SHY, AY, APR, SCCA, SCCA ratio)

The digestion of MS at 14 d HRT in both reactors was influenced by the reactor start-up and the one-time base addition, leading to increased rates of hydrolysis and acidogenesis and to steadily decreasing pH- and conductivity measurements. However, in PFR1 the establishment of the microbial community can be seen by stable conversion rates and yields and the stabilization of the FDAP measurement in weeks 7 and 8 (see Fig. 1). The FDAP measurement determines the cell polarizability and thereby reflects the physiological state of the mixed culture. Increases in FDAP (and concomitantly in the transmembrane potential of cells) are associated with a higher activity, growth and hydrolysis while decreases might indicate stressed conditions (e.g., acidic stress) with low microbial activity [40]. Digestion of MS at 14 d of HRT in PFR1 was characterized by relatively high acid production and acidification (81%), yielding in particular butyric acid (72%) and acetic acid (17%) among the analyzed SCCAs with an average hydrolysis of 23.5%. MS digestion in PFR2 at 14 d of HRT with recirculation also led to dominant butyric acid synthesis with a share of 70% of butyric acid and 18% of acetic acid among the SCCAs (see Additional file 1: Fig. S1). Methane production was not observed (≤ 0.1%), due to the low pH-value between 4.4 and 4.1. High fluctuations in the gas production were detected, in which maximal production occurred after feeding events with a decreasing trend until the next feeding. The application of recirculation increased the gas production by 35% to 836.9 mL kg_VS_^−1^ and reduced the daily fluctuations significantly (see Additional file 1: Figs. S1 and S2).

The shift of the HRT from 14 to 7 d in week 9 induced major changes in PFR1 (Fig. 1). The concentration of soluble metabolites was reduced by half (sCOD) or more (SCCA, red. sugars). A shorter HRT thus induced washout, higher stress of the microbial community as indicated by a decreasing FDAP measurement and reduced hydrolysis by a quarter (to 17%) and acidification by one-fifth (to 65%). While the SCCA concentrations were reduced, their proportional composition remained rather stable, with a slight increase of propionic acid. The significant increase of conversion yields in the first two weeks after the change of the HRT is mainly caused by the metabolite washout. Nevertheless, the shorter HRT of 7 d caused a significant increase in the SHR that stabilized in the 3rd week (that is after more than 2 exchanges of the liquid volume). It was more than twice as high as compared to conditions when a HRT of 14 d was applied. Correspondingly, also the SHY at 7 d of HRT was slightly higher (431 g_O2_ kg_VS_^−1^, + 11%) compared to an HRT of 14 d. The APR was increased less, resulting in a slightly reduced AY of 174 g_SCCA_ kg_VS_^−1^ (− 14%).

To determine the effect of thin-sludge recirculation, 20% of the harvest was recirculated while holding 7 d of HRT in PFR1 during the following experimental phase. Based on similar metabolite concentrations and rates in weeks 11 and 12, it is likely and assumed that a quasi-steady state was achieved in the third HRT for both conditions at an HRT of 7 d. Thin-sludge recirculation increased and stabilized the soluble metabolite concentrations, hydrolysis (19%) and acidification (74%, see also Fig. 4). A higher share of pyruvic and citric acid among all SCCAs was measured. This can be an effect of the improved availability of nutrients and side-products, which might increase fluxes through the tricarboxylic acid cycle. The direct effect of recirculation can best be seen by the immediate increase of the FDAP at the inlet, while microbes at the center and outlet ports take longer to reach the same status (Fig. 1, left). Recirculation improved cell viability, likely by a better availability of nutrients and substrate, higher enzyme concentration and increased digestion time of soluble polymers. Here, the FDAP measurement proved to be a fast indicator of changing process conditions that are relevant for the cell physiology. However, only small effects of recirculation on the conversion rates were found.

The effect of recirculation on the digestion of MS was also tested at an HRT of 14 d in PFR2 (in Additional file 1: Fig. S1, Table S4), however no comparable quasi-steady state was reached without recirculation, as the reactor was still influenced from the start-up conditions.

### Straw digestion—impact of the hydraulic retention time

In order to investigate the hydrolysis capacity of a more recalcitrant feedstock in the PFRs, 30% or 66% of MS were replaced with bedding straw. As the straw features a higher content of hard-to-digest lignocellulosic structures, 14 d and an extended HRT of 21 d were tested. 20% of thin-sludge recirculation was constantly applied to investigate its effect on the process performance. Both PFRs were operated with 30% and 66% straw, respectively: while in PFR1 bioaugmentation experiments were conducted as described in detail elsewhere [38], the effect of HRT was examined in PFR2.

**Fig. 2:**
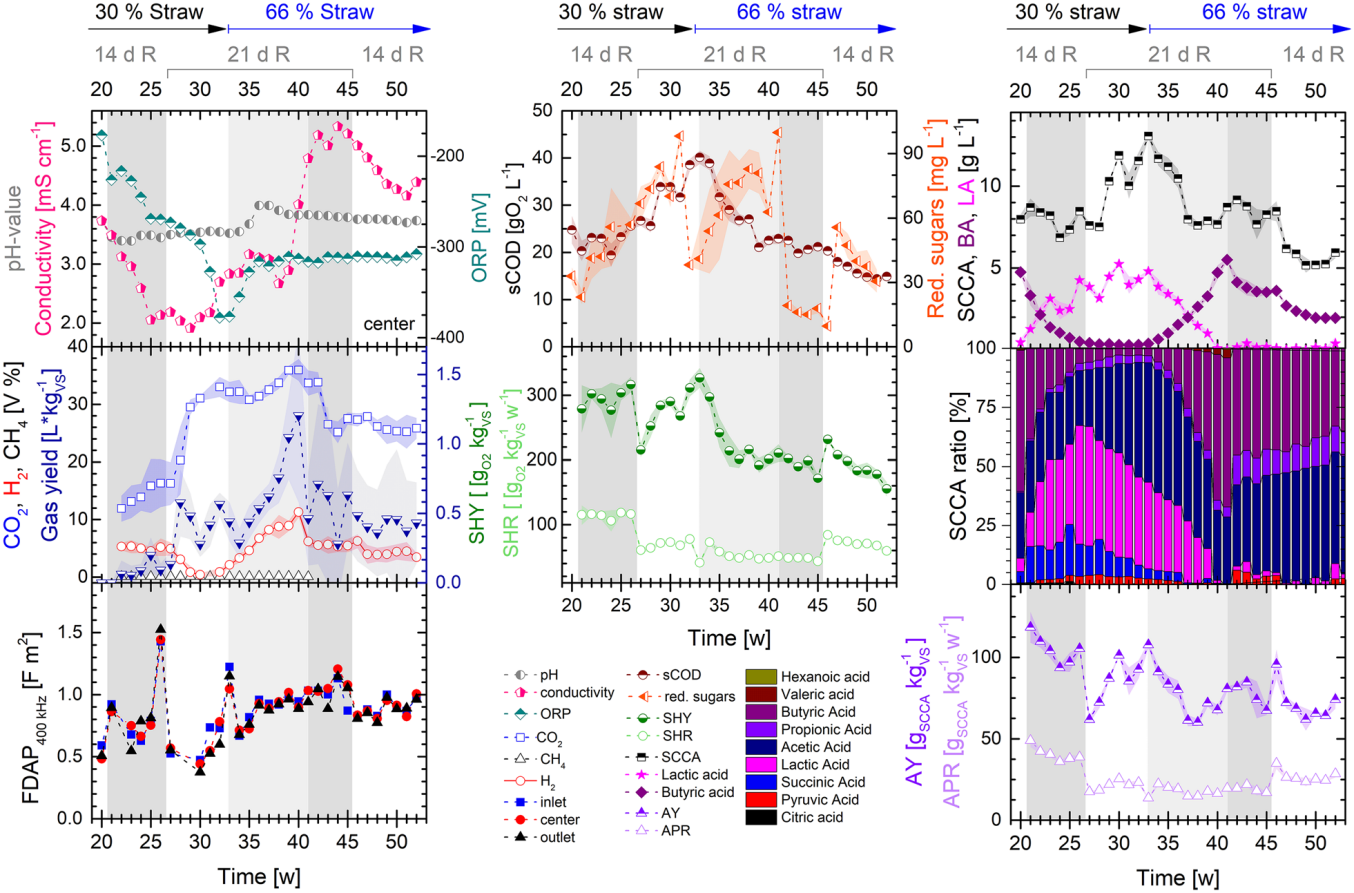
Process conditions in PFR2 operating with MS mixed with bedding straw at 30% and 66% ratio with different HRT (14 d/21 d) and 20% of recirculation (R). Left: *On-line* monitoring of pH, conductivity and ORP at the center port of the PFR, *on-line* measurement of the gas flow and composition and FDAP measured at 400 kHz. Center: measurements of sCOD, total reducing sugars (center), SHY and SHR. Right: total SCCA concentration, butyric acid (BA), lactic acid (LA), composition of the SCCA fraction, AY and APR. Depicted are the average values of *on-line/off-line* measurements of a week from each port of the PFR (inlet, center, outlet) or the average over all ports of the PFR with shaded deviations between ports (SHR, SHY, AY, APR, SCCA, BA, LA, SCCA ratio). Different metabolic phases during the digestion at 21 d of HRT with 66% straw (w/w) are marked with shades of grey

The digestion of 30% straw (w/w) in PFR2 started in week 20 and was directly operated in a continuous manner with 14 d of HRT from week 21 on. During this reactor start-up, the pH-value dropped below 3.5 (see Fig. 2). This led to a metabolic shift of the butyrate producing microbial community towards higher shares of lactic (42%), acetic (26%) and succinic acid (18%) within the measured SCCA. After 2 HRTs of operation with 30% of straw (w/w), the culture reached a quasi-steady state with a hydrolysis of 32% and an acidification of 36%. Compared to MS, the application of 30% straw as feedstock showed higher hydrolysis, but considerably lower acidification. An increased content of suspended solids and a reduced particle size of residual substrate could be confirmed visually compared to the butyric type fermentation. Under the same feedstock loading rates with 30% straw in PFR1, an acidification of 55% was achieved with a dominance of butyric acid accumulation (see Table 3 and [38]). In PFR2, a steady gas production of 145.7 mL kg_VS_^−1^ with a share of 16.1% CO_2_ and 4.9% of H_2_ was detected.

In comparison to an HRT of 14 d, a longer HRT of 21 d significantly increased the concentration of soluble metabolites and the CO_2_ production. The accumulation of lactic acid up to 5 g L^−1^ was accompanied by the depletion of butyric acid and its associated hydrogen production by week 30. The accumulation of acetic acid increased at the HRT of 21 d up to 6.6 g L^−1^, while the lactic acid concentration fluctuated between 3.1 and 5.2 g L^−1^. However, the SHY and AY decreased to 290 g_O2_ kg_VS_^−1^ and 89 g_SCCA_ kg_VS_^−1^, respectively, showing that the longer HRT rather led to metabolite accumulation and gas production, but did not increase the specific conversion yields or rates of hydrolysis or acidogenesis from the recalcitrant feedstock (see also Fig. 4). Cell polarizability decreased from 1124 (HRT of 14 d) to 859 F m^2^ (HRT of 21 d), likely due to higher acid stress from acid accumulation. An increasing trend of the total reducing sugars can be seen after the substrate changed to 30% and to 66% of straw, and after the HRT changed from 21 to 14 d with 66% of straw. Changes of the dominant microbial metabolism, e.g., from lactic to butyric acid production or vice versa (weeks 20 to 26 and 35 to 41) led to the accumulation of reducing sugars, most likely due to the slow adaption processes in the microbial community and a temporarily reduced substrate uptake.

A substrate change from 30 to 66% of straw (w/w), while maintaining an HRT of 21 d led to a lower metabolite concentration, reduced conversion yields and rates (SHR decreased by one-third), a higher pH-value around 3.8, and to an increased FDAP of 1035 F m^2^. Subsequently, the microbial community switched back towards dominant butyrate production with the depletion of lactic acid accumulation. Butyrate production started straight after the change of the feedstock composition. Within the adaption period of the microbial community marked in light grey in Fig. 2, the SHY and AY sink before weeks 40/41. Gas production and hydrogen concentration increased concurrently with the butyric acid concentration until weeks 40/41, before stabilizing at a lower level. A stable acidogenic fermentation of 66% of straw (w/w) is achieved in the last HRT (weeks 43 to 45) with a hydrolysis of 29% and acidification of 59%, producing mainly butyric (48%), acetic (35%) and propionic acid (10%) at a pH-value of 3.8 and ORP of − 310 mV.

A lower HRT of 14 d with 66% straw (w/w) feedstock further reduced metabolite concentrations and decreased the gas production, CO_2_ and H_2_ concentrations. However, the conversion yields for hydrolysis and acidogenesis remained within a similar range due to increased SHR and APR. This fits with the observations with 30% straw, namely that a higher HRT leads to metabolite accumulation and gas production and not to increased hydrolysis (see also Fig. 4). The FDAP decreased to about 880 F m^2^, although acid stress was obviously decreased. This could be an indicator for reduced metabolic activity of the microbial community due to substrate deficiency and insufficient hydrolysis of the recalcitrant straw. Increasing conductivity measurements during the digestion of 66% of straw (w/w) could be related to an increased manure content in the feedstock between weeks 31 and 45. In contrast to straw, animal manure is rich in nutrients and ionic compounds which can increase its basal conductivity and thereby the conductivity measurements in the liquid phase.

### Straw digestion—impact of recirculation

The effect of thin-sludge recirculation on mixed straw digestion (66% straw) was further investigated by the application of 10 and 20% recirculation of the outflow in comparison to no recirculation in PFR1.

**Fig. 3:**
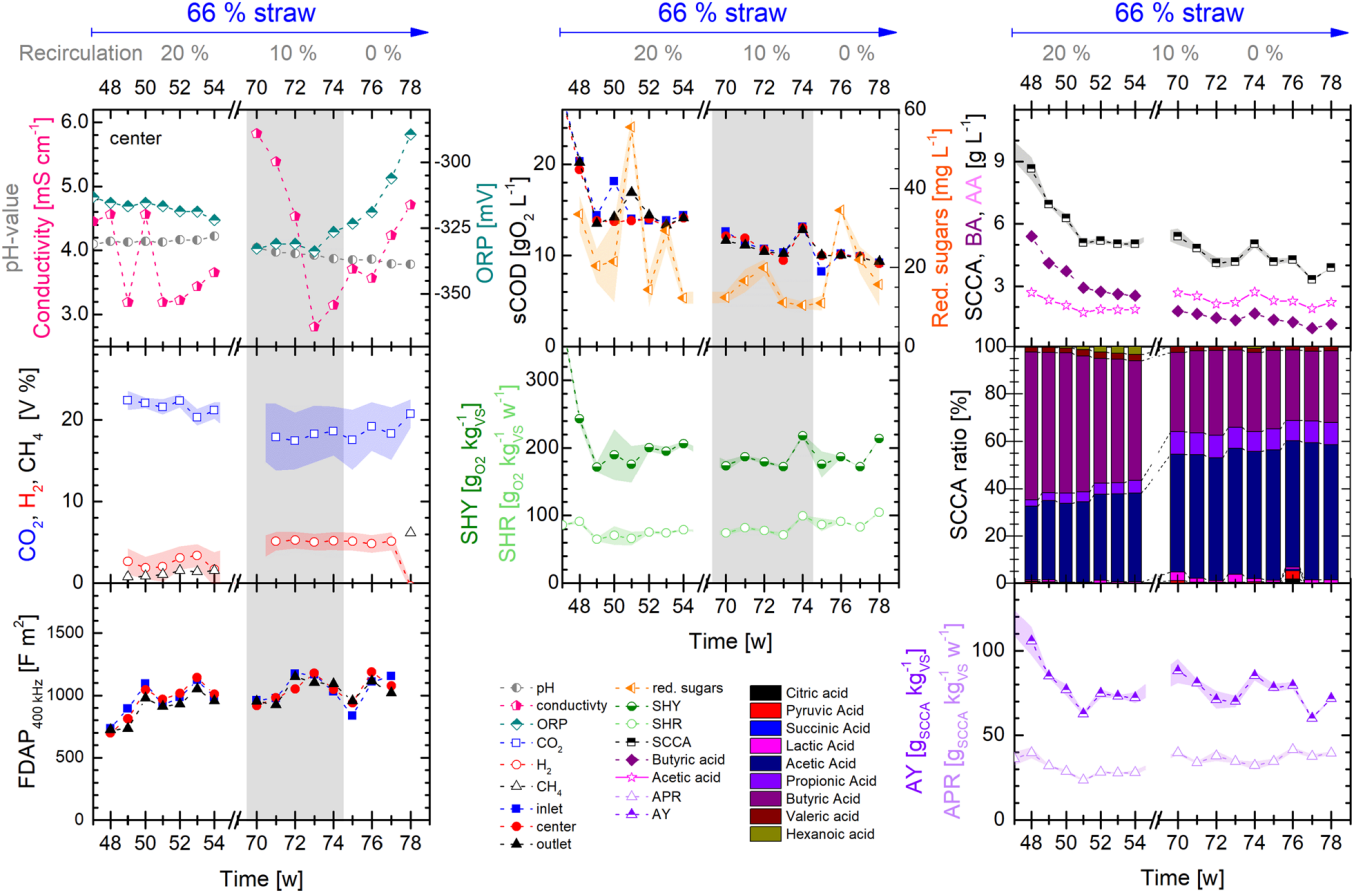
Process conditions in PFR1 operating with 66% (w/w) bedding straw mixed with MS at different recirculation ratios (0/10/20%). Left: *on-line* monitoring of pH, conductivity and ORP at the center port of the PFR, *on-line* measurement of the gas flow and composition and FDAP measured at 400 kHz. Center: measurements of sCOD, total reducing sugars (center), SHY and SHR. Right: total SCCA concentration, butyric acid (BA), acetic acid (AA), composition of the SCCA fraction, AY and APR. Depicted are the average values of *on-line/off-line* measurements of a week from each port of the PFR (inlet, center, outlet) or the average over all ports with shaded deviations between ports (SHR, SHY, AY, APR, SCCA, BA, AA, SCCA ratio)

**Fig. 4:**
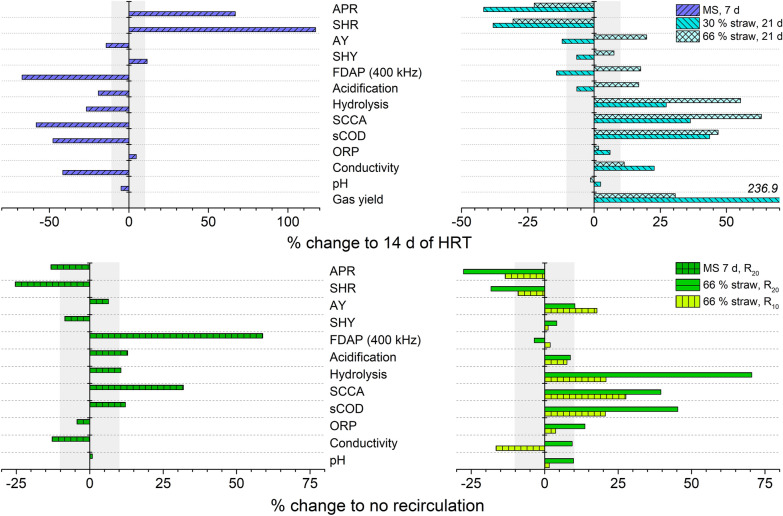
Percentual change of process parameters in quasi-steady-state conditions for a changed HRT (top) and thin-sludge recirculation (bottom). 7 d of HRT was tested with MS (left), 21 d of HRT were tested with 30% and 66% of straw (w/w, right) and all compared to 14 d of HRT of the corresponding feedstock. 10–20% of thin-sludge recirculation were applied in the digestion of 66% straw (w/w, right) at 14 d HRT and MS at 7 d HRT (left). In case of recirculation, the corresponding operation w/o recirculation served as reference. A change below 10% is marked in gray as it is considered to be marginal and related to dynamic fluctuations rather than altered process conditions

Recirculation increased metabolite concentrations, hydrolysis and decreased fluctuation in the gas production (Fig. 3). While hardly any effect could be seen on the SHY, the AY was increased with recirculation, where a 10% recirculation showed a maximum increase of 18% compared to the non-recirculated condition (see Fig. 4). The SCCA profile showed a long-term (30 weeks) adaption process when a higher straw content was applied. The SCCA production stabilized with a dominant accumulation of acetic acid (54%) along with butyric (32%) and propionic acid (9%) among all SCCA. Changing recirculation did not show a positive effect on the conversion rates of SHR and APR.

### Comparability of parallel reactor experiments

**Fig. 5:**
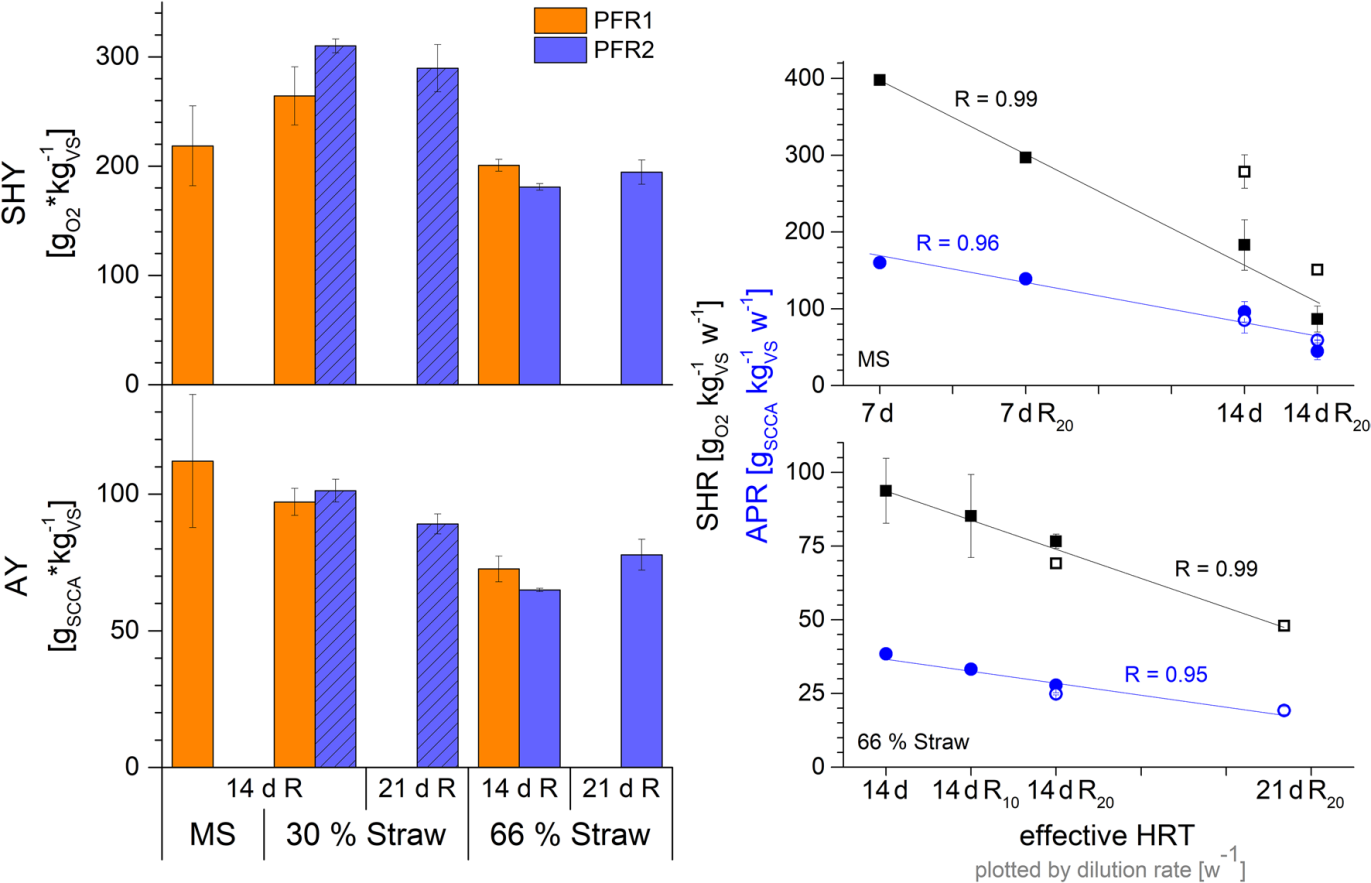
Left: comparison of the average conversion yields between the parallel reactors at quasi-steady-state conditions depending on feedstock and HRT. The application of thin-sludge recirculation of 20% is indicated by R. Crosshatched columns indicate lactic acid type fermentation, solid columns a butyric type fermentation. Right: dependance of the specific hydrolysis rate (SHR, black squares) and the acid production rate (APR, blue circles) on the effective dilution rate in the PFRs for MS (top) and 66% straw (bottom). Filled symbols present PFR1, open symbols PFR2. Linear correlation was found depending on the feedstock, the Pearson correlation coefficient is displayed. The data of PFR2 during MS digestion (before shut-down) were not included in the linear correlation

Experiments in this publication were conducted in two parallel reactors to i) verify reproducibility of digestion tests and ii) test multiple conditions in a shorter time frame. Hydrolytic digestion of all feedstocks was conducted at an HRT of 14 d as shown in Fig. 5. For the mixed straw substrates, the parallel reactors show a good agreement of the conversion yields. The AY is very similar with 97–101 and 65–73 g_SCCA_ kg_VS_^−1^ for 30% and 66% of straw correspondingly. A little higher deviation than the standard deviation at quasi-steady state was found for the SHY, ranging from 264–309 and 180–200 g_O2_ kg_VS_^−1^ for 30% and 66% of straw, respectively. The different acidogenic pathways, which were directed either to butyric or lactic acid, did not affect the conversion yields significantly during the digestion of 30% straw, as it only led to a slightly increased SHY. Higher deviations in the conversion yields between reactors can be found for the digestion of MS. These are majorly the effect of a higher total solids concentration in PFR2, and thus a higher substrate availability. Normalizing the conversion yields to the solids concentration showed that (1) SHY was increased by a higher solids content, likely due to higher enzyme–substrate interactions, and (2) AY was very similar for both PFR, and thus dependent on substrate availability (see Additional file 1: Table S5). While higher SHRs were reached in PFR2 due to higher substrate load, the APRs were comparable to PFR1, showing that acidogenic conversion was very similar in both reactors. The rates of hydrolysis and acidogenesis show a linear dependence on the dilution ratio in the PFR (see Fig. 5), which is applicable for both reactors. Thin-sludge recirculation decreased the dilution rate and thus increased the effective HRT, as parts of the harvest were recirculated and the water input decreased. Since the recirculated conditions fit well within the rate-dependance on the dilution ratio, the decreases of conversion rates with recirculation (as shown in Fig. 4) are assumed to be minor and only due to a higher effective HRT. This data shows that the evaluation of process conditions at quasi-steady state under the applied, dynamic process conditions is reliable, also across the two different reactor cultivations as conducted in this study.

## Discussion

The suitability of a PFR as a hydrolysis stage for residual biomass was successfully demonstrated without further pretreatment or pH-control. Hydrolytic and acidogenic digestion can be improved by the application of suitable process conditions, namely the HRT and thin-sludge recirculation, among others, which was examined in this study.

### Effect of HRT and recirculation

In acidogenic digestion, the HRT has to be sufficient to allow substrate solubilization and conversion to acids, but should not support growth of methanogens and still be reasonable in terms of throughput [25]. All tested feedstock compositions showed good digestion efficiency at 14 d of HRT. Increases in HRT resulted in decreased conversion rates of hydrolysis and acidogenesis for all feedstock (see Figs. 4, 5). Linear correlations of the SHR and APR to the effective HRT were found for MS and 66% straw. The SHR is higher at shorter HRT, probably due lower acid concentrations and a lower re-assimilation by microbes. The drastic decrease of FDAP measurements at 7 d of HRT with MS digestion indicates that the microbial community was stressed under these conditions, possibly leading to long-term adaptations of it. The lower slope of the APR at short HRT (7 d of HRT, see Fig. 5) indicates a reduction of the acidogenic performance. This might be caused by the partial washout of soluble compounds and finally also of acidogenic microorganisms.

The overall hydrolysis yield (SHY) was not improved by a longer HRT. Most likely, readily degradable polymers such as starch (in MS) or hemicellulose were degraded at all HRTs, whereas degradation of recalcitrant crystalline cellulose-lignin structures was limited by the low pH-value, as it was described for various cellulolytic microorganisms [41–43]. However, with the mixed straw feedstock, significantly increased gas production (+ 237% with 30% straw) might have led to an underestimation of the SHY. The AY was increased at a longer HRT, especially with a higher content of recalcitrant feedstock (+ 20% with 66% straw). Contrary effects of the HRT on AY were found with 30% straw, possibly due to the different microbial activity with a preferred lactic acid production. The increase of the AY in parallel with the HRT was described before for the acidogenic digestion of kitchen waste [22, 23, 44], food waste [24] and the co-digestion of primary and waste activated sludge [21].

Thin-sludge recirculation increased the AY by 6–18% and led to metabolite accumulation in the liquid phase (e.g., SCCA increase by 28–40%, see Fig. 4). Similar results were found by Luo et al*.*, who described that recirculation increased acid production and acidification [2]. No significant effect of the recirculation was found on the SHY. The conversion rates of hydrolysis and acidogenesis were reduced with recirculation, however this is mostly due to the higher effective HRT under recirculation conditions. If the rates are related to the same effective HRT, virtually no effect of recirculation on SHR was found, while increased recirculation slightly reduced the APR. This is presumably caused by product inhibition due to a higher SCCA concentration and higher acidification (+ 8–13%) in the PFR.

The ORP showed an increasing trend with recirculation during MS digestion, but a rather opposite trend during the digestion with 66% of straw. It is believed, that the changing ORP level is rather related to long-term changes in the microbial community. For example, a stabilization of the ORP at − 310 mV was found after the switch to a dominant butyric acid production. Especially at the short HRT of 7 d with MS digestion, recirculation improved the cell viability by 59% as measured with FDAP. This could indicate a higher stability of the microbial culture—also shown by a steady gas production—and possibly bacterial enrichment, meaning that a high SHR and APR under short HRT might be achievable on long term under recirculation. A positive effect of recirculation on the FDAP has also been found during bioaugmentation experiments in the same reactor system [38]. Enrichment of hydrolytic and acidogenic bacteria through recirculation in a PFR was previously described by Dong et al. [9].

Increasing the HRT and the recirculation can thus increase the AY from recalcitrant substrate. However, both HRT and thin-sludge recirculation, have a minor effect on the hydrolysis yield under acidic conditions. A shorter HRT is feasible without a loss of the hydrolysis efficiency and leads to a lower acid content. While recirculation had a lower effect on the conversion rates than the HRT, it likely had a stabilizing effect on the microbial community.

### Dynamics of SCCA accumulation

With all feedstocks and under all operation conditions, both PFRs operated as hydrolytic-acidogenic digestion stage, characterized by the accumulation of SCCA and a CO_2_ and H_2_ release. Two distinct fermentation profiles were found in dependence of the pH-value: (1) a dominant butyric acid production and (2) a dominant lactic acid production.

Usually butyric acid production in acidogenic AD is found only at higher pH-values between 5.0 and 6.3 [16, 19]. At pH-values between 3.4 and 4.4, as obtained in this study, butyric acid appears mostly in its undissociated form that can disturb the intracellular pH-value [45], leading to acidic stress as confirmed by FDAP measurements. However, butyric acid producing bacteria were active and dominant until pH 3.8, where usually a shift towards a dominance of lactic acid bacteria was described in other studies [19, 46, 47]. The higher concentration of recalcitrant carbohydrates provides an advantage for butyric acid producing bacteria like *Clostridia* spp. [19, 42, 48]. Moreover, thin-sludge recirculation might have helped to maintain a stable microbial community. A balance between butyric acid- and lactic acid-producing communities is typical for the acidic fermentation of carbohydrates, where a pH below 4.0 leads to dominance of lactic acid bacteria and lower biodiversity [16, 19]. Most often, butyric and acetic acid along with hydrogen are the desired products from acidogenic AD while lactic acid is undesired, also because of its inhibiting effect on *Clostridia* spp. [15]*.* To prevent accumulation of lactic acid in the PFR-system, a pH-drop below 3.5, especially during start-up, and a high OLR featuring high amounts of easily degradable substrate should be avoided. However, pH-values below 4.0 were not necessarily found to inhibit butyrate producing bacteria, if the butyric acid producing microbial community was established, and further stabilized by recirculation or eventually by evolved microenvironments in the PFRs.

Being able to control the production spectrum of a hydrolytic phase is of high interest, as different products can be obtained in dependence of the subsequent processes. For our reactors, a stable butyric acid dominance among the SCCA fraction can be obtained by maintaining the pH-value above 3.8 with recirculation. Butyric (and acetic) acid can be used as substrate for methanogenesis or in other microbial processes. As it has been shown in this study, the acidogenic metabolism did not significantly influence the acidogenesis yield, so both products were obtained with similar efficiency. The lower pH-value during lactic acid fermentation might even contribute to higher hydrolysis, as seen in our study, due to acidic disruption of the lignocellulosic structures [49, 50].

### Digestion efficiency

Digestion of MS in the PFRs in our study reached a SHY between 218 and 395 g_O2_ kg_VS_^−1^ and an AY from 112 to 203 g_SCCA_ kg_VS_^−1^ (see Table 3). Higher SHY was found for a higher content of TS as in PFR2 and a shorter HRT, while acidogenesis was increased with longer HRT and thin-sludge recirculation. Similar results for SHY and AY of 204 g_sCOD_ kg_VS_^−1^ and 183 g_SCCA_ kg_VS_^−1^ in a shorter HRT of 4 d were found by Cavinato et al*.* [51] in the acidic co-digestion of MS with cow manure in a stirred tank reactor and controlled pH of 5.5. Benito Martin et al*.* [52] reached a SHY^1^ of 393 g_sCOD_ kg_VS_^−1^ and AY^1^ of 291 g_SCCA_ kg_VS_^−1^ in the acidogenic digestion of MS at controlled pH 5.0–5.5 at an HRT of 17 d. Acidogenic digestion of corn straw silage in a leach bed reactor at controlled pH of 8.0 resulted in high acid yields of 310 g_SCCA_ kg_COD_^−1^ [53]. High acidification of 60–81% during MS digestion in our study indicates that lower yields were due to limited hydrolysis rather than to low acidogenesis. The optimum pH-value for hydrolytic bacteria is mostly within pH 5.0 and 7.0 [43], whereas a lower pH-value can severely inhibit the growth and enzyme production of especially *Clostridium* spp. [41, 54, 55]. Nevertheless, yields in our study are comparable with literature values.

Similar results were found in both PFRs for the mixed straw substrates, when SHY and AY decreased with the increasing content of straw. In our study, a maximum acid production of 77.6–77.8 g kg_VS_^−1^ was achieved in both reactors with 66% of bedding straw. Yield calculations from a hydrolysis stage of straw are rare and thus hard to compare. In the thermophilic digestion of rice straw, acid accumulation of 3 g L^−1^ in a continuous process [33] or 6.0–8.3 g_COD_ L^−1^ during batch digestion [56] were reached. Here, a concentration of about 5 g L^−1^ of SCCA was reached with 66% of straw under mesophilic conditions at 14 d HRT.

The bedding straw used in this study contained varying amounts of horse manure, however the nitrogen content as measured in the feedstock was still quite low. Higher manure content can improve buffering and nutrient balance in the PFR and by this increase hydrolysis [36, 57, 58]. Hence, co-digestion with nitrogen-containing feedstock is a further strategy to stabilize the process.

The PFR system is not meant to be a stand-alone process, but a hydrolytic stage for consecutive biorefinery systems. Therefore, digestion efficiency in the second stage would be greatly improved by the microbial and acidic pre-digestion, loosening the lignocellulosic structure in the PFR. This concept has been validated by Motte et al. [32]: a dark fermentation process was used as a combined biologic and acidic pretreatment of wheat straw followed by mechanical milling and a subsequent stage for bioethanol production from the pretreated substrate. The application of such a concept resulted in a 35% lower energy demand for the combined pretreatment than just milling and a higher overall substrate conversion to bioethanol and SCCA of 131% compared to the non-digested straw.

## Conclusion

The effects of HRT and thin-sludge recirculation during plug-flow based digestion of MS and MS mixed with bedding straw were examined. Shorter HRT increased the conversion rates of hydrolysis and acidogenesis, but did not improve the hydrolysis yield. Hydrolysis of lignocellulose in the PFR was limited by the low pH-values between 3.4 and 4.3, and thus rather affected by this than the HRT and thin-sludge recirculation. Recirculation showed lower quantitative effects on the hydrolytic digestion than HRT, but is assumed to have positively influenced the stability of the microbial community at low pH-value and a short HRT of 7 d. Both operation parameters had a strong influence on the metabolite concentrations, e.g., the SCCA concentrations were increased by up to 45.3% or 63.1% through recirculation or HRT variation. Further increase of the conversion yields could be reached by operation at higher pH-values, achievable, for instance, by thin-sludge recirculation from a second methanogenic stage in a two-stage digestion.

## Supplementary Information

**Additional file 1**. Supplementary material.

## Data Availability

The datasets used during the current study are available from the corresponding author on reasonable request.

## Abbreviations

AD: Anaerobic digestion
AY: Acid yield
APR: Acid production rate
FDAP: Frequency-dispersed polarizability anisotropy
HRT: Hydraulic retention time
MS: Maize silage
ORP: Oxidation–reduction potential
PFR: Plug-flow reactor
sCOD: Soluble chemical oxygen demand
SHY: Specific hydrolysis yield
SHR: Specific hydrolysis rate
SCCA: Short-chain carboxylic acids
tCOD: Total chemical oxygen demand
TS: Total solids
TVS: Total volatile solids
VS: Volatile solids

## Footnotes

1 Metabolic yields were calculated by the authors of this manuscript upon available data.
