# Supplementary material for: Plug-flow hydrolysis with lignocellulosic residues: effect of hydraulic retention time and thin-sludge recirculation
Source: Biotechnol Biofuels Bioprod. 2023 Jul 6;16:111. doi: 10.1186/s13068-023-02363-7 (PMC10326939; doi:10.1186/s13068-023-02363-7)
Supplement: Supplementary file 1 — Additional file 1. Supplementary material. [file 13068_2023_2363_MOESM1_ESM.pdf]

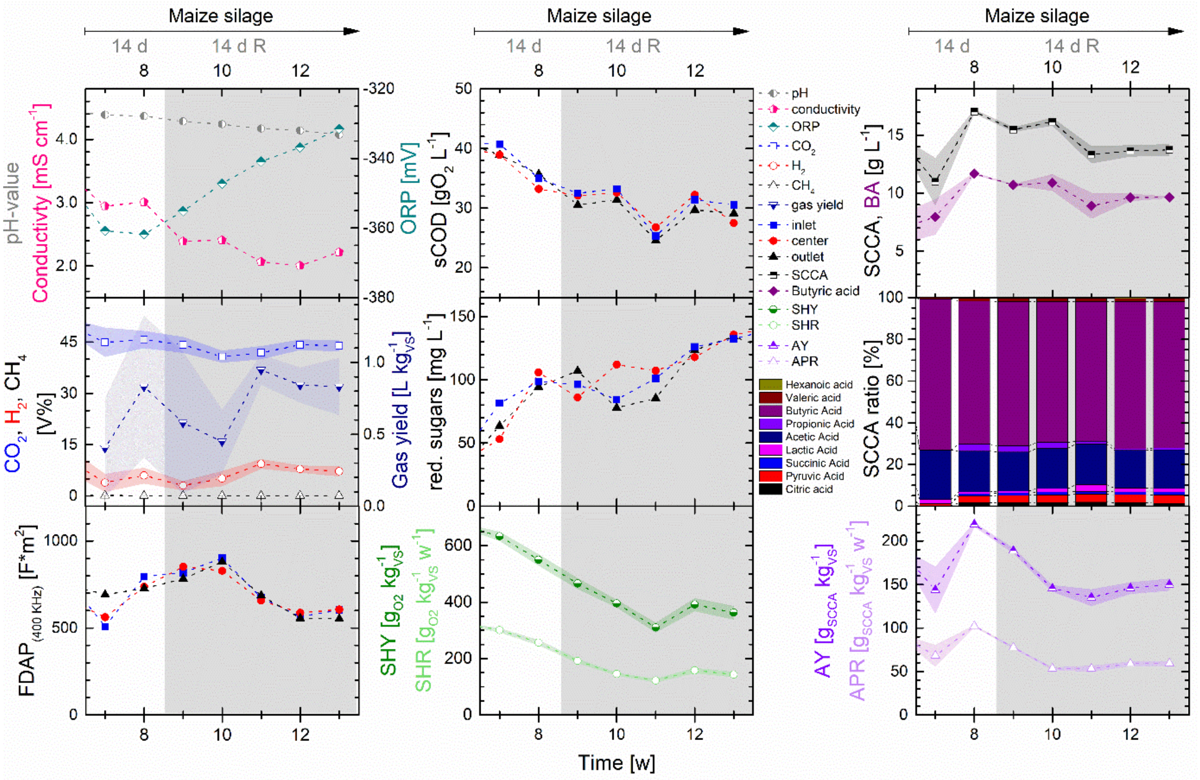

Figure S1 Process conditions in PFR2 operated with maize silage at 14 d HRT with and w/o 20 % recirculation. The dynamic start-up phase and the one time pH-addition influenced the digestion until week 10. Left: *On-line* monitoring of the pH-value, conductivity and ORP measured at the center of the PFR, *on-line* measurement of the gas flow and composition and FDAP measured at 400 kHz; Center: measurements of sCOD, total reducing sugars, SHY and SHR; Right: total SCCA concentration, butyric acid (BA), composition of the SCCA fraction, AY and APR. Depicted are average values of *on-line/off-line* measurements gained during a week from each port of the PFR (inlet, center, outlet) or the average values over all ports with shaded deviations in between ports (SHR, SHY, AY, APR, SCCA, BA, SCCA ratio).

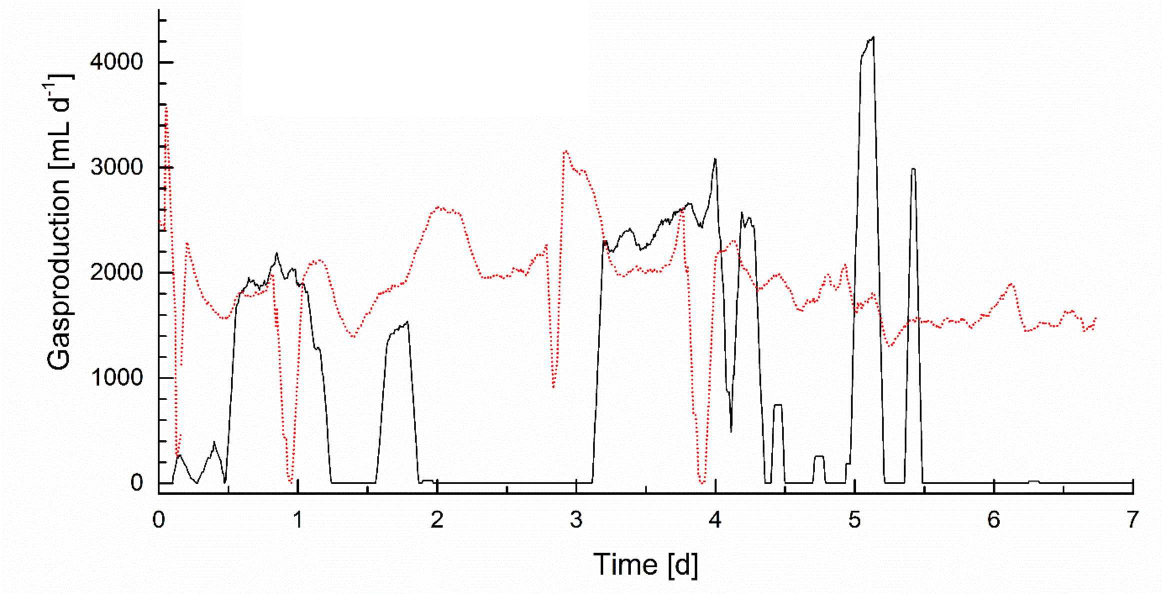

Figure S2 Example of the fluctuations of gas production over one week: Hourly average of gas production in PFR2 without recirculation (week 7, black solid lines) and with 20 % recirculation (week 12, red dotted line). Feeding occurred during the days 0, 1, 3 and 4, also visible by production drops as the gas output from pressure compensation during feeding was subtracted from the net gas production.

Table S3 Content characterization of all substrate batches used in the experiments

	Parameter	Unit	*Whole plant maize silage*				
Period of operation	*PFR1*	w	1-3	4 - 12	13 - 25	26 - 45	45 -78
	PFR2		/	1 - 6	7 - 16	20 - 31	32 - 53
	pH		3.73 (0.014]	6.04 (0.03)	3.77 (0.01)	5.91 (0.08)	3.75 (0.04)
	Total solids	%	37.65 (0.47)	28.20 (0.43)	26.47 (8.31)	25.69 (1.06)	27.61 (0.52)
	Moisture content	%	62.35 (0.47)	71.80 (0.43)	73.53 (8.31)	74.31 (1.06)	72.39 (0.52)
	Volatile solids	% of TS	97.46 (0.03)	97.39 (0.49)	97.44 (0.06)	95.78 (0.32)	96.16 (0.14)
	Ashes	% of TS	2.54 (0.03)	2.61 (0.49)	2.56 (0.06)	4.22 (0.32)	3.84 (0.14)
	tCOD	$gO_2\ g_{TS}^{-1}$	0.88 (0.02)	0.81 (0.08)	0.87 (0.04)	0.81 (0.06)	0.88 (0.06)
	sCOD	$gO_2\ g_{TS}^{-1}$	0.098 (0.00)	0.11 (0.03)	0.12 (0.00)	0.16 (0.01)	0.18 (0.01)
	Total nitrogen	% of TS	0.92 (0.06)	1.09 (0.18)	0.81 (0.02)	0.92 (0.02)	0.99 (0.04)
	Non-structural Carb. content	% of TS	33.30 (1.456)	47.60 (3.38)	35.6 (15.9)	23.6 (2.69)	17.92 (3.43)
	Acid insoluble lignin	% of TS	13.50 (1.78)	13.82 (0.27)	13.64 (1.94)	17.13 (0.38)	17.27 (0.75)
	Acid soluble lignin	% of TS	1.12 (0.02)	0.84 (0.03)	1.51 (0.02)	1.40 (0.02)	2.53 (0.02)
	Lactic acid	% of TS	/	/	/	/	3.27
	Succinic acid	% of TS	/	/	/	/	0.92

	Parameter	Unit	*Bedding straw with horse manure*				
Period of operation	*PFR1*	w	31 - 44	45 – 52	52 -59	59 - 68	68 – 78
	PFR2		20 - 30	31 -37	38 -44	45 - 53	/
	pH		/	6.45 (0.08)	8.09	8.62 (0.01)	7.37 (0.45)
	Total solids	%	/	/	/	/	/
	Moisture content	%	/	/	/	/	/
	Volatile solids	% of TS	87.23 (4.02)	90.47 (1.08)	70.94 (10.82)	93.87 (1.93)	75.21 (17.2)
	Ashes	% of TS	12.77 (4.02)	9.53 (1.08)	48.60 (10.82)	6.13 (1.93)	24.79 (17.2)
	tCOD	$gO_2\ g_{TS}^{-1}$	0.23 (0.03)	0.33 (0.06)	1.15 (0.10)	0.27 (0.05)	0.53 (0.16)
	sCOD	$gO_2\ g_{TS}^{-1}$	0.07 (0.0)	0.08 (0.00)	0.03 (0.01)	0.03 (0.00)	0.07 (0.01)
	Total nitrogen	% of TS	0.36 (0.04)	0.70 (0.12)	0.96 (0.00)	0.47 (0.19)	0.60 (0.02)
	Non-structural Carb. content	% of TS	16.27 (0.30)	11.00 (4.26)	9.86 (0.99)	5.65 (4.33)	21.05 (1.08)
	Acid insoluble lignin	% of TS	27.25 (2.98)	30.32 (1.08)		26.44 (1.35)	32.74 (1.32)
	Acid soluble lignin	% of TS	1.15 (0.08)	2.15 (0.01)		1.14 (0.06)	0.95 (0.02)

Table S4 Average values of digestion of MS at 14 d of HRT in PFR2 during weeks 7/8 without reaching a quasi-steady state

Parameter	Unit	Values
sCOD	$g_{O2}\ L^{-1}$	37.1 ±2.4
SCCA	$g_{SCCA}\ L^{-1}$	14.0 ± 3.0
Acetic acid	%	21.6 ± 2.1
Butyric acid	%	70.5 ± 1.9
Lactic acid	%	1.45 ± 0.6
Hydrolysis	%	30.4 ± 2.0
Acidification	%	61.9 ± 17.0
SH	$g_{O2}\ kg_{VS}^{-1}$	591.9 ± 41.9
AY	$g_{SCCA}\ kg_{VS}^{-1}$	185.8 ± 37.7

Table S5 Average SH and acid production of parallel reactors digesting MS in relation to their corresponding total solids content

	SH /TS $[\frac{g_{O_2}}{kg_{VS}*\%_{TS}}]$		Acid production / TS $[\frac{g_{SCCA}}{kg_{VS}*\%_{TS}}]$	
	14d	14d R	14d	14d R
PFR1	25.2 ± 0.4	17.5 ± 2.9	13.6 ± 1.1	9.0 ± 1.9
PFR2	41.0 ± 13.7	26.0 ± 1.0	13.5 ± 2.8	10.2 ± 0.1
